# Introduction

**DOI:** 10.1155/S1463924691000019

**Published:** 1991

**Authors:** Peter B. Stockwell


					Journal of Automatic Chemistry, Vol. 13, No. (January-February 1991), p. 1
Special Issue: International Symposium on
Laboratory Automation and Robotics 1990.
Management Papers
Volume 13, Number 1, is .devoted to papers presented at the eighth International Symposium on
Laboratory Automation and Robotics, which was held in Boston, USA from 16 to 19 September
1990. The issue includes five papers on Managing Automation and Robotics, plus the conference's
plenary lecture by Glenn Taylor et al., and Francis Zenie (President of the Zymark Corporation) on
his view of the future for robotics.
A full report on the meeting was published in Journal ofAutomatic Chemistry, Vol. 12, No. 6, together
with the abstracts of all the papers and posters presented at the Symposium.
The papers in this issue are reprinted, with permission, from the Proceedings ofthe 1990 International
Symposium on Laboratory Automation and Robotics. I would like to thank all those at the Zymark
Corporation, especially Sharon Correia, who have made this special issue possible.
Readers might wish to note the dates for the ninth International Symposium on Laboratory
Automation and Robotics" 20 to 23 October 1991 at the Boston Park Plaza Hotel. For details about
papers, posters or registrations readers should contact Christine O'Neill, Zymark Corporation,
Zymark Center, Hopkinton, Massachusetts 01748, USA (tel." 508 435 9500).
CONTENTS
Robotics fulfil a strategic need, G. L. Taylor, T. R. Smith and G. J. Kamla, p. 3.
Robotic applications" lessons on what constitutes success, B. Hutchins, p. 9.
Managing robotics in the bioanalytical/metabolic environment of a pharmaceutical company,
S. Kushinsky, p. 13.
Challenges facing modern automated laboratories' robotic applications, E. A. Elnenaey, J. Pluscec
and V. Fernandez, p. 17.
Managing an automation development group, S. D. Hamilton, p. 23.
Six years of robots, G. F. Plummer, G. Waterworth and W. Roberts, p. 29.
Vision of the 80s- Reality of the 90s!, F. H. Zenie, p. 39.
PETER B. STOCKWELL

				

